# Wnt2 Contributes to the Development of Atherosclerosis

**DOI:** 10.3389/fcvm.2021.751720

**Published:** 2021-11-24

**Authors:** Jinyu Zhang, Samuel Rojas, Sanjay Singh, Phillip R. Musich, Matthew Gutierrez, Zhiqiang Yao, Douglas Thewke, Yong Jiang

**Affiliations:** ^1^Department of Biomedical Sciences, James H. Quillen College of Medicine, East Tennessee State University, Johnson City, TN, United States; ^2^Division of Infectious, Inflammatory and Immunologic Diseases, Department of Internal Medicine, Quillen College of Medicine, East Tennessee State University, Johnson City, TN, United States; ^3^Department of Biological Sciences, College of Arts and Sciences, East Tennessee State University, Johnson City, TN, United States; ^4^Department of Health Sciences, College of Public Health, East Tennessee State University, Johnson City, TN, United States

**Keywords:** TGF-β, EndMT, atherosclerosis, Wnt, HAECs

## Abstract

Atherosclerosis, is a chronic inflammatory disease, characterized by the narrowing of the arteries resulting from the formation of intimal plaques in the wall of arteries. Yet the molecular mechanisms responsible for maintaining the development and progression of atherosclerotic lesions have not been fully defined. In this study, we show that TGF-β activates the endothelial-to-mesenchymal transition (EndMT) in cultured human aortic endothelial cells (HAECs) and this transition is dependent on the key executor of the Wnt signaling pathway *in vitro*. This study presents the first evidence describing the mechanistic details of the TGF-β-induced EndMT signaling pathway in HAECs by documenting the cellular transition to the mesenchymal phenotype including the expression of mesenchymal markers α-SMA and PDGFRα, and the loss of endothelial markers including VE-cadherin and CD31. Furthermore, a short hairpin RNA (shRNA) screening revealed that Wnt2 signaling is required for TGF-β-mediated EndMT of HAECs. Also, we found that LDLR^−/−^ mice fed on a high-fat western-type diet (21% fat, 0.2% cholesterol) expressed high levels of Wnt2 protein in atherosclerotic lesions, confirming that this signaling pathway is involved in atherosclerosis *in vivo*. These findings suggest that Wnt2 may contribute to atherosclerotic plaque development and this study will render Wnt2 as a potential target for therapeutic intervention aiming at controlling atherosclerosis.

## Introduction

Atherosclerosis is characterized by thickening of the arterial wall plaque resulting in narrowing of the vessel and restricted blood flow. Atherosclerotic plaques arise from the accumulation of immune cells, smooth muscle cells (SMC), fibroblasts, lipids and extracellular matrix (ECM) in the arterial wall. These atherosclerotic changes are the major cause of heart attacks, peripheral vascular disease and other illnesses that collectively account for the majority of deaths and morbidity in the world (1, 2). The atherosclerotic risk factors including high blood pressure, smoking and obesity result in disturbing the blood flow. This suggests the importance of haemodynamic forces in the initiation of the disease (3). Several recent studies have suggested that a link between the endothelial-to-mesenchymal transition (EndMT) and the activation of endothelial TGF-β signaling may be an important driver of atherogenesis (4, 5). Although both EndMT and TGF-β signaling play roles in atherosclerosis, the mechanistic link between TGF-β and EndMT in atherosclerotic disease progression remains obscure.

Previous studies demonstrated that mesenchymal cells originate from endothelial cells through EndMT (6). EndMT makes a major contribution to the vascular remodeling and neointimal formation that arises following vein graft transplantation into the arterial circulation^17^. This process is dependent on early activation of the Smad2/3-Snai2 signaling pathway, with antagonism of TGF-β signaling resulting in decreased EndMT and reduced neointimal formation. Chen et al. (7) extended the understanding of EndMT and demonstrated that it promotes atherosclerosis progression. This report showed that the extent of EndMT strongly correlates with the extent of atherosclerosis and inversely correlates with expression of fibroblast growth factor receptor 1 (FGFR1) (7). However, the principal mesenchymal cell type that is derived from EndMT in atherosclerosis was not defined in their study, nor was the extent of the contribution of EndMT-derived mesenchymal cells addressed. Mesenchymal cells are essential to the progression of atherosclerosis since they secrete proinflammatory molecules and synthesize extracellular matrix proteins and metalloproteases that promote plaque buildup and modulate the stability of plaques (8). Moreover, endothelial dysfunction plays a critical role in the development of atherosclerosis (9, 10). As is well-known, EndMT, as a type of endothelial dysfunction, is characterized by the cellular phenotypic changes including the detachment and migration away from the endothelium, the loss of endothelial properties and a gain of mesenchymal properties. The latter include enhanced proliferation and migration and secretion of extracellular matrix proteins with the expression of stem cell markers such as stem cell antigen 1 (Sca1) and zinc finger protein (Snai1), mesenchymal markers such as α-smooth muscle actin (α-SMA), and the extracellular matrix proteins collagen and platelet-derived growth factor receptor α (PDGFRα) (7, 11, 12). In addition, non-laminar flow induces tumor growth factor (TGF)-β signaling (13) and promotes the process of EndMT *in vitro* and *in vivo* (7, 14–16), acting by induction of Snai1 (17).

Mesenchymal cells including fibroblasts are prominent in atherosclerosis, with fibroblast-specific proteins being highly expressed in advanced atherosclerotic lesions (18, 19). Further studies have identified that EndMT-derived fibroblast-like cells are common in intimal atherosclerotic plaques using both Cre-lox endothelial lineage tracking in mice, and in human plaques by detecting cells co-expressing endothelial and fibroblast/mesenchymal proteins indicative of EndMT. Therefore, based on the fact that atherosclerosis is an archetypal example of a TGF-β-mediated fibrosing vascular disease of adults (20, 21), we elected to investigate how TGF-β-induced EndMT contributes to atherosclerotic plaque formation using a cell culture model *in vitro* and low density lipoprotein-deficient (LDLR^−/−^) mice fed a high fat western (HF) diet *in vivo* model. Together, these experimental models reveal insights into the mechanisms controlling TGF-β-induced EndMT that are relevant to atherosclerotic plaque development and are important in guiding the development of new therapies aimed at reversing vascular remodeling to reduce atherosclerosis.

## Materials and Methods

### Cell Culture and Reagents

Human aortic endothelial cells (HAECs), purchased from the American Type Culture Collection (ATCC, Manassas, VA, United States), were grown in complete Endothelial Cell Medium (ECM) supplemented with endothelial growth factors along with 5% FBS and 1% penicillin/streptomycin. All cells were maintained at 37°C in a humidified atmosphere of 5% CO_2_.

### Western Blotting

Western blot (WB) analyses were performed as previously described (22). Total cellular proteins were separated by loading a specified amount of whole cell lysate (WCL) on a denaturing 8–15% polyacrylamide gel, followed by transfer to polyvinylidene difluoride (PVDF) membranes. The membranes were blocked with a 5% non-fat milk solution and incubated with primary antibodies for Wnt2, VE-cadherin, CD31, PDGFRα, αSMA (Cell Signaling Biotechnology) or Hsp90 (Santa Cruz Biotechnology). Secondary antibodies (Thermo Fisher Scientific) conjugated to horseradish peroxidase and ECL (Bio-Rad) were used to detect signals which were visualized by chemiluminescence.

### Real Time-Quantitative PCR

Total RNA was extracted with the RNeasy® plus Mini Kit (Qiagen) and cDNA was synthesized from 1 μg of RNA using High-Capacity cDNA Reverse Transcription Kits (Applied Biosystems). SYBR Gene Expression Assay Mix and Fast Universal PCR Master Mix (Applied Biosystems) were employed to measure the expression of target genes. Real-time PCR was performed using the 7,900 HT Fast system (Applied Biosystems). The expression of a target gene was calculated relative to the expression of β-actin or GAPDH.

#### Immunofluorescence Staining

For immunofluorescence, cells cultured on coverslips or Optimal cutting temperature (OCT)-embedded frozen atherosclerotic lesions sections were fixed in 4% paraformaldehyde for 15 min, permeabilized with 0.1 % Triton X-100 for 15 min and blocked with 3% BSA (all in in PBS) for 30 min. Then, cells on coverslips were incubated at 4°C overnight with primary antibodies and then with Alexa Fluor 488-labeled anti-mouse or Alexa Fluor 594-labeled anti-rabbit secondary antibodies (Abcam). Cells on coverslips or frozen sections were mounted with mounting solution with DAPI (Vector Laboratories, 1:500). Confocal fluorescence images were taken with a Leica TCS SP8 confocal microscope.

### Immunohistochemistry

Tissue sections were deparaffinized, rehydrated by different grade ethanol, and heated in Antigen Retrieval Citra Solution, pH 6. Sections were incubated with Dual Endogenous Enzyme Block solution (Dako, Glostrup, Denmark), and were incubated with Protein Block Serum-Free solution (Dako). Slides were then incubated with a mouse monoclonal anti-wnt2 antibody (Santa Cruz; dilution, 1:100) for overnight at 4°C. The signals were detected using the Vectastain Elite ABC kit (Vector Laboratories, Burlingame, CA) following the manufacturer's protocol. Hematoxylin was used for counterstaining.

### Atherosclerosis Lesion Analysis

Mice after 12 weeks with a western diet were sacrificed and perfused through the heart with 10 mL PBS. Segments of tissue (~1 cm cubes), with or without fixation in 4% formaldehyde/PBS, were embedded in OCT freezing medium (Thermo Scientific) and immediately frozen on dry ice. Samples were stored at −80°C until used. Eight μm cryostat sections of the aortic sinus were applied onto poly-L lysine-coated slides (Sigma) and stained with Oil Red O and hematoxylin.

### Statistical Analysis

All results are expressed as the mean ± SEM or SD. Mice and sample groups were *n* > 3, unless otherwise indicated. Data were analyzed using the unpaired student *t*-test, and treatment differences were considered significant at *p* value <0.05. Kaplan-Meier survival curves were generated using GraphPad Prism software.

## Results

### TGF-β2 Promotes EndMT

**Figure 1:**
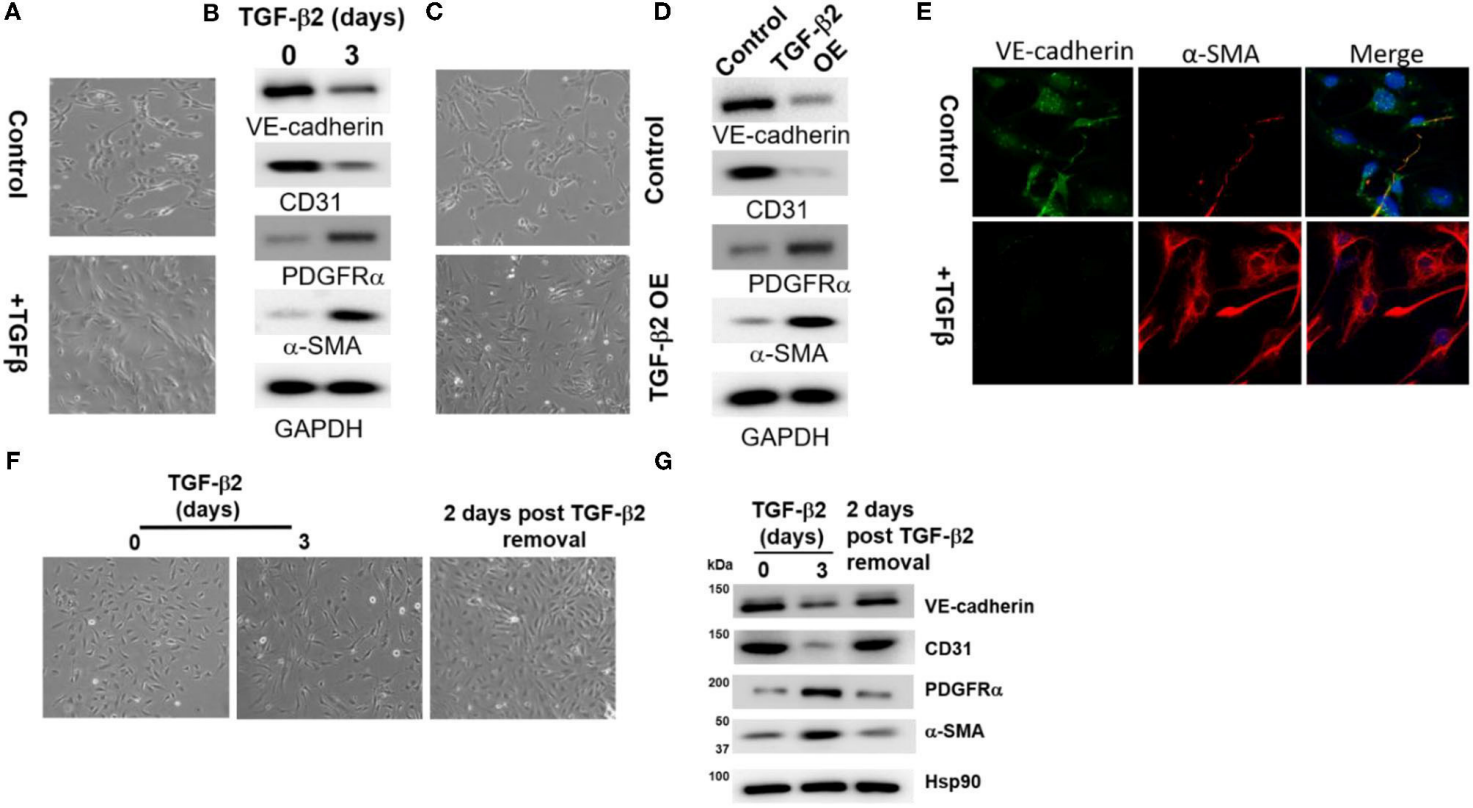
TGF-β2 induces EndMT in HAECs. **(A)** The morphological change of HAECs before and after a 3-day TGF-β2 treatment. Scale bars, 100 μm. **(B)** WB detection of the expression of the EndMT markers VE-cadherin, CD31, PDGFRα, and αSMA. Hsp90 was used as a loading control. **(C,D)** Same experiments were performed as those in **(B,A)** to control HAECs and TGF-β2-overexpressing HAEC cells. **(E)** Immunostaining of VE-cadherin and αSMA in control HAECs and HAECs treated with TGF-β2 for 3 days. Scale bars, 100 μm. Experiments were repeated three times. **(F)** The morphological change of HAECs 2 days post TGF-β2 removal following a 3-day TGF-β2 treatment. **(G)** Immunoblotting for the expression of the EndMT markers VE-cadherin, CD31, PDGFRα, and αSMA in HAECs for **(F)**. Hsp90 was used as a loading control.

When treated with TGF-β2, HAECs show a distinct change from cobblestone-like endothelial cell morphology to a fibroblast-like, spindle-shaped morphology (Figure 1A). WCL from TGF-β2-treated cells were analyzed by WB to detect the expression of the endothelial cell markers, VE-cadherin and CD31, and mesenchymal markers, PDGFRα and α-SMA. VE-cadherin and CD31 levels decreased in cells treated with TGF-β2, while PDGFRα and α-SMA levels increased (Figure 1B). Similarly, HAECs stably overexpressing (OE) lentivirus-delivered TGF-β2 exhibited a mesenchymal fibroblast phenotype compared to control cells infected with empty vector (Figures 1C,D). The complementary assay of HAEC cells with or without TGF-β2 treatment by immunofluorescence microscopy showed similar results (Figure 1E). Since atherogenesis is a chronic process, we also exposed the HAEC cells to TGF-β2 for a longer time point (5 days) as shown in Supplementary Figure 1, we observed that the EndMT status is still maintained, which is confirmed by the expression of EndMT markers. Interestingly, after the TGF-β was removed from cell culture media followed 3 days treatment, the cells reverted from mesenchymal phenotype to epithelial phenotype that is demonstrated by the cellular morphology and EndMT markers (Figures 1F,G).

### TGF-β2 Attenuates Cell Invasion in EndMT *in vitro* Model

**Figure 2:**
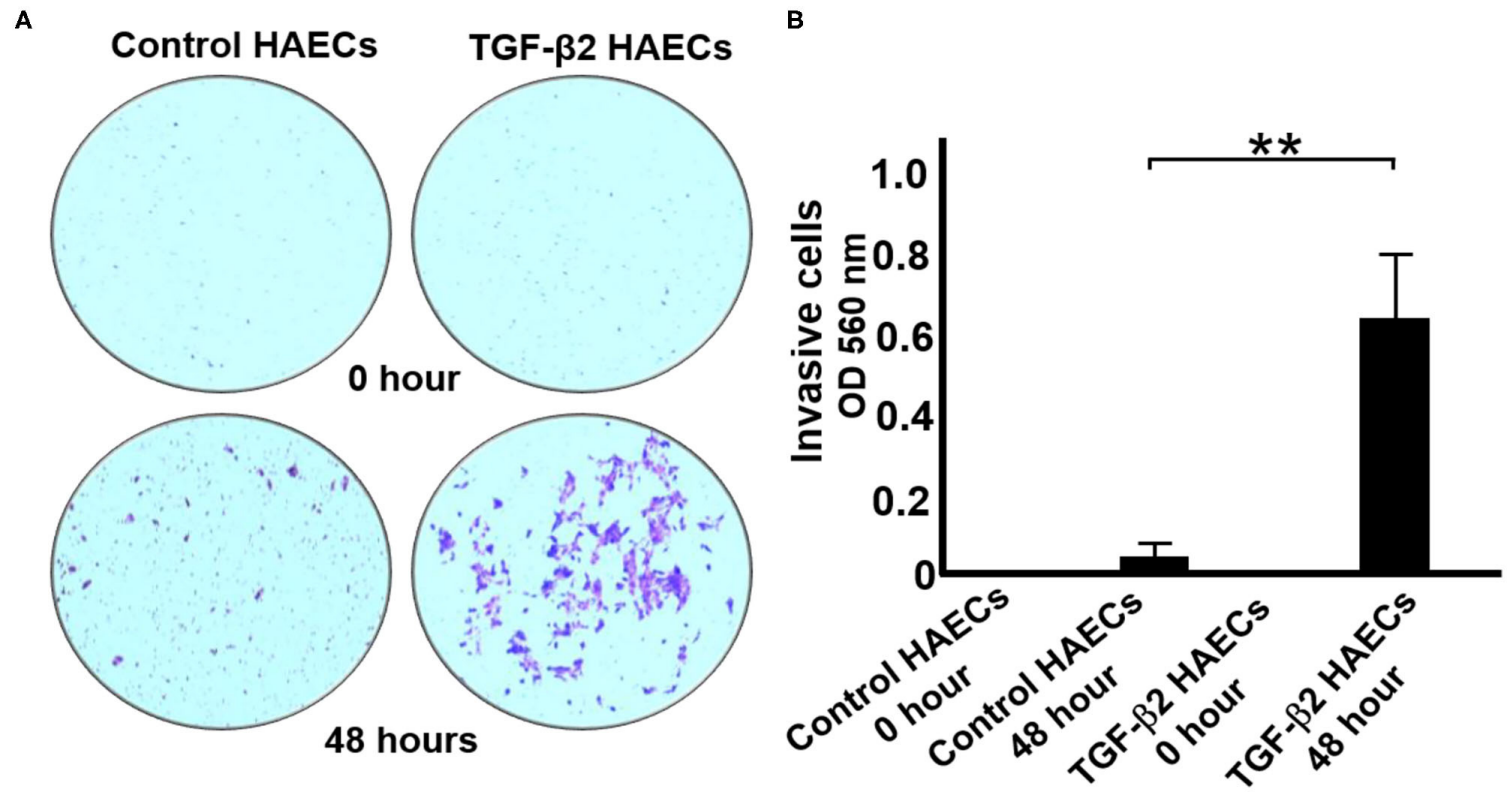
A cell invasion assay of the effect of TGF-β2 treatment of HAECs. **(A)** The comparison of invasive capabilities between control HAECs and TGF-β2-overexpressing HAECs. Top: The cells on the bottom of the basement membrane before the invasion (0 h). Bottom: The cells on the bottom of membrane after 48 h of invasion. **(B)** The quantification of cell invasion assays in **(B)**. *n* = 3, **P < 0.01.

We performed cell invasion assays to determine if there was a change in the invasive potential of HAECs following EndMT employing a commercially available 24-well Matrigel cell invasion assay kit (Cell Biolabs, Inc.). Figure 2 shows a comparison of invasive capability between normal and TGF-β2-overexpressing HAEC cells. We observed that after 48 h a great number of TGF-β2-overexpressing HAECs attached to the bottom of basement membrane, whereas few control HAECs were observed on the bottom of basement membrane (Figures 2A,B). These results indicate that TGF-β2 induces EndMT in HAECs and, following EndMT, these cells acquire the migratory and invasive capabilities associated with the mesenchymal phenotype.

### Induction of Wnt Ligand Expression During TGF-β2-Mediated EndMT

**Figure 3:**
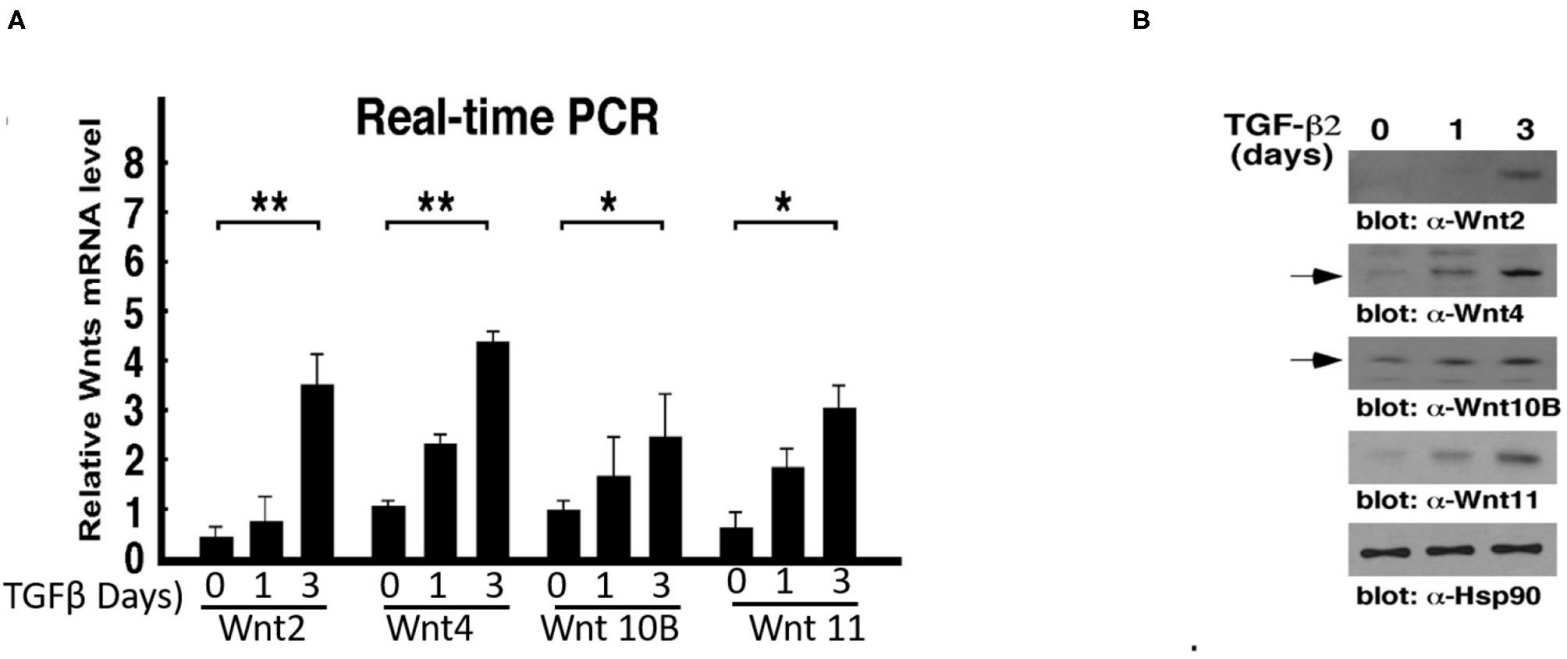
Wnt ligand expression is upregulated during TGF-β-mediated EndMT. **(A)** RT-PCR analysis of Wnt2, Wnt4, Wnt10B, and Wnt11 mRNA expression during TGF-β-mediated EndMT for different time courses. Each value represents the mean ± SD of three separate experiments. n = 3, *P < 0.05, **P < 0.01. **(B)** WB analysis of Wnt ligand protein expression during TGF-β-mediated EndMT. WCLs collected from cells treated with TGF-β2 for 0, 1, and 3 days were subjected to WB analysis. Hsp90 protein expression is used as a loading control.

We next investigated the molecular mechanisms underlying endothelial lineage specification of fibroblast-like cells during EndMT. While its role in EndMT is essentially unknown, prior studies have documented that TGFβ/Smad3 and Wnt pathways promote vascular smooth muscle cell proliferation during the related process of the epithelial-to-Mesenchymal transition (EMT). Thus, we speculated that Wnt may be of relevance during the EndMT occurring during the formation of atherosclerotic plaques. To determine how the Wnt signaling pathway plays an important role in TGF-β2-mediated EndMT, we isolated RNA from HAECs treated with TGF-β2 for 0, 1, and 3 days, and performed RT-PCR to analyze the expression of Wnt family members with their corresponding specific primers. The quantitative analysis demonstrated the induction of Wnt2 (4.5-fold), Wnt4 (5.6-fold), Wnt10B (2.5-fold) and Wnt11 (5.3- fold) after EndMT (Figure 3A). The immunoblot analysis of Wnt protein expression is consistent with our PCR analysis (Figure 3B). The results indicated that there was a significant upregulation in both the mRNA expressions and protein levels of Wnt2, Wnt4, Wnt10B, and Wnt11 after TGF-β2 treatment.

### TGF-β2 Induced EndMT Is Wnt2-Dependent

**Figure 4:**
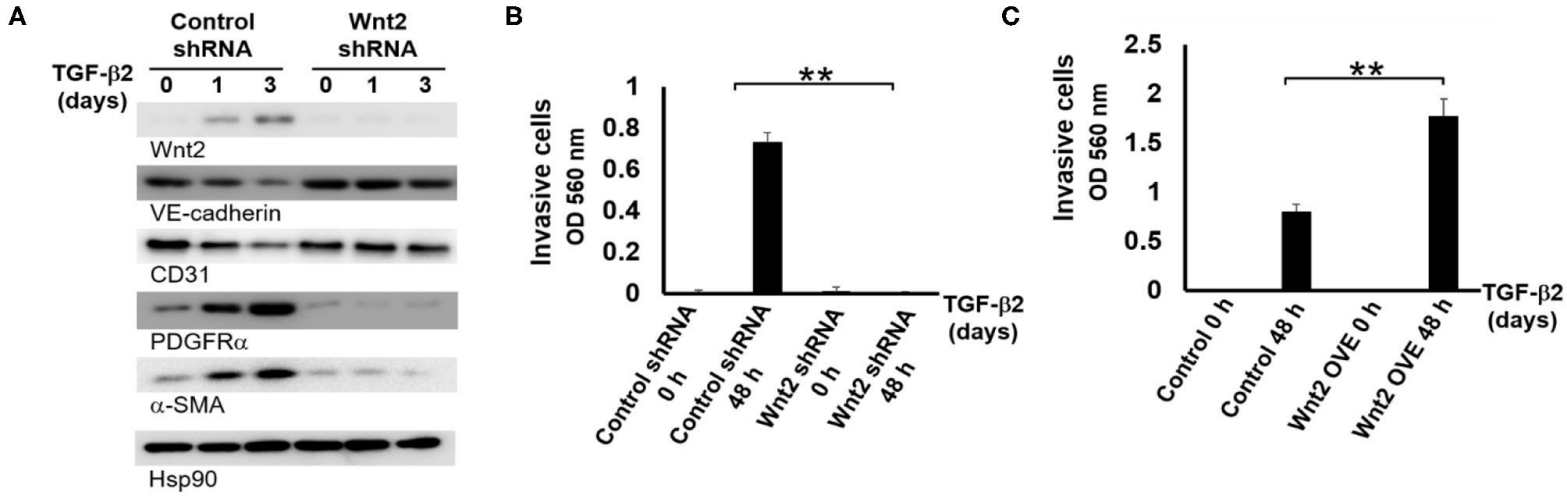
Wnt2 is required the TGF-β2 mediated-EndMT in cells migration and invasion. **(A)** HAECs stably transfected with control scrambled shRNA and Wnt2 shRNA were treated with TGF-β2 over time as indicated in the figure. Then, the WCLs were subjected to WB analysis. VE-cadherin, CD31, PDGFRα, and α-SMA. Hsp90 expression was used as a loading control. Experiments were repeated three times. **(B)** The comparison of invasive capabilities between control and Wnt2 shRNA with TGF-β2 treatment HAECs in the indicated time. Each value represents the mean ± SD. *n* = 3, **P < 0.01 **(C)** The comparison of invasive capabilities between control and Wnt2 overexpression (OVE) with TGF-β2 treatment HAECs in the indicated time. Each value represents the mean ± SD. *n* = 3, **P < 0.01. **(D**) Immunoblotting analysis of the EndMT markers in Wnt4 KD, Wnt10B KD, and Wnt11 KD HAECs with or without TGF-β2 treatment for 3 days, respectively. WCLs collected from KD cells treated with TGF-β2 for 0 and 3 days were subjected to immunoblots using the indicated antibodies. Hsp90 protein level is used as a loading control. Experiments were repeated three times.

We employed shRNA technology to identify which Wnt protein functions to regulate TGF-β2-induced EndMT. We found that only the silencing of Wnt2 could significantly attenuate TGF-β2-mediated EndMT. In control HAECs transfected with scrambled shRNA, TGF-β2 increased the expression of the mesenchymal markers PDGFRα and α-SMA, and decreased the endothelial markers VE-cadherin and CD31. In shRNA-mediated Wnt2 knockdown (KD) HAECs, TGF-β2 failed to induce the expression of PDGFRα and α-SMA (Figure 4A), However, shRNA-mediated Wnt4, Wnt 10B, or Wnt11 KD in HAECs doesn't significantly attenuate TGF-β2-induced EndMT, which is shown by the levels of EndMT protein markers (Supplementary Figure 2). In addition, Wnt2 silence attenuated the invasive capability of HAECs (Figure 4B) and Wnt2 overexpression (OVE) promoted the invasive capability of HAECs (Figure 4C). Together, our results indicate that Wnt2 facilitates TGF-β2-mediated EndMT.

### The Role of Wnt2 Is Determined in High Fat-Induced Atherosclerosis

**Figure 5:**
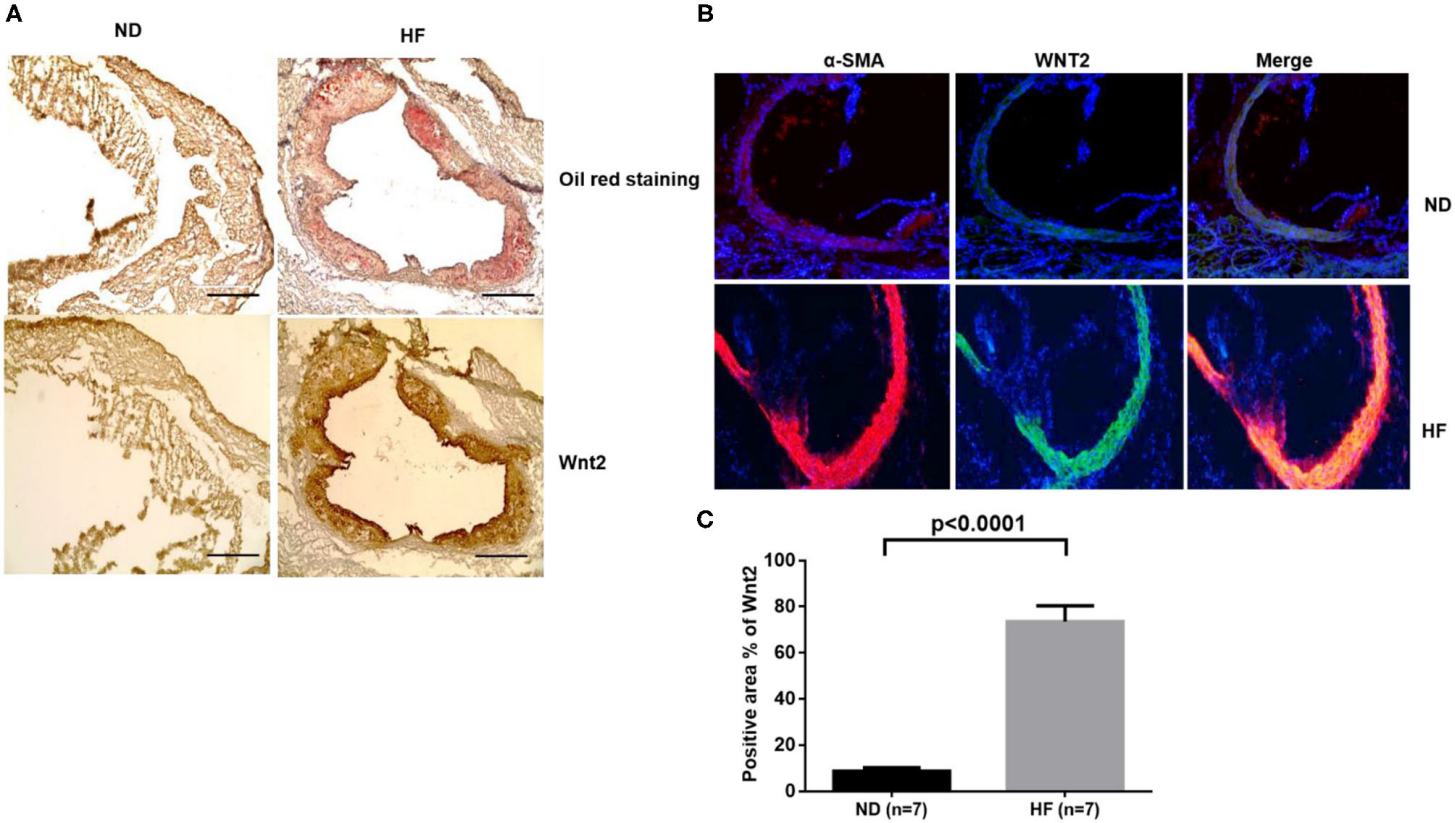
Wnt2 is involved in the atherosclerotic lesion development. Upper panels **(A)**: Oil Red O staining of atherosclerotic lesions in the aortic root at the level of the aortic valves of LDLR^−/−^ mice fed on a normal diet (ND) and a high-fat (HF) diet. Lower panels: Immunohistochemistry staining of Wnt2 protein. Magnification is x10, scale bars = 400 μm. **(B)** Immunostaining for α-SMA (Red) and Wnt2 (Green) expressions in atherosclerotic lesions derived from the aortic valves of LDLR^−/−^ mice fed on a normal diet (ND) and a high-fat (HF) diet. **(C)** Quantification for the Wnt2 staining area in α-SMA-positive atherosclerotic lesions derived from the aorta. Each value represents the mean ± SD. *n* = 7.

Recently, emerging evidence suggests that in diabetic LDLR^−/−^ mice, an ectopic BMP2-Msx2 gene regulating Wnt signaling is upregulated in association with vascular calcification (23). Here, we confirmed that the aorta had an increased lesion area in LDLR^−/−^ mice fed a HF diet (21% fat, 0.2% cholesterol) compared to mice fed a normal diet. Aorta were sectioned and stained with Oil Red O for lipids, counterstained with hematoxylin, and digitally photographed. Figure 5 shows a representative Oil Red O-stained aortic root section (Figure 5A, upper panel). In addition, we detected an increase in Wnt2 protein by immunohistochemical staining of the aorta from the LDLR^−/−^ mice fed a high-fat western-type diet compared to those mice fed a normal diet (Figure 5A, lower panel). This data indicates that Wnt2 proteins are enriched in the area of the atherosclerotic lesion. To further confirm whether these Wnt2 positive areas are related to the cells undergone EndMT, we co-stained Wnt2 and EndMT marker α-SMA by immunostaining. We found a significant increase positive area of the colocalization between Wnt2 and α-SMA at 70% in the atherosclerotic lesions derived from the LDLR^−/−^ mice fed with a high-fat western-type diet when compared to those mice fed with a normal diet.

## Discussion

**Figure 6:**
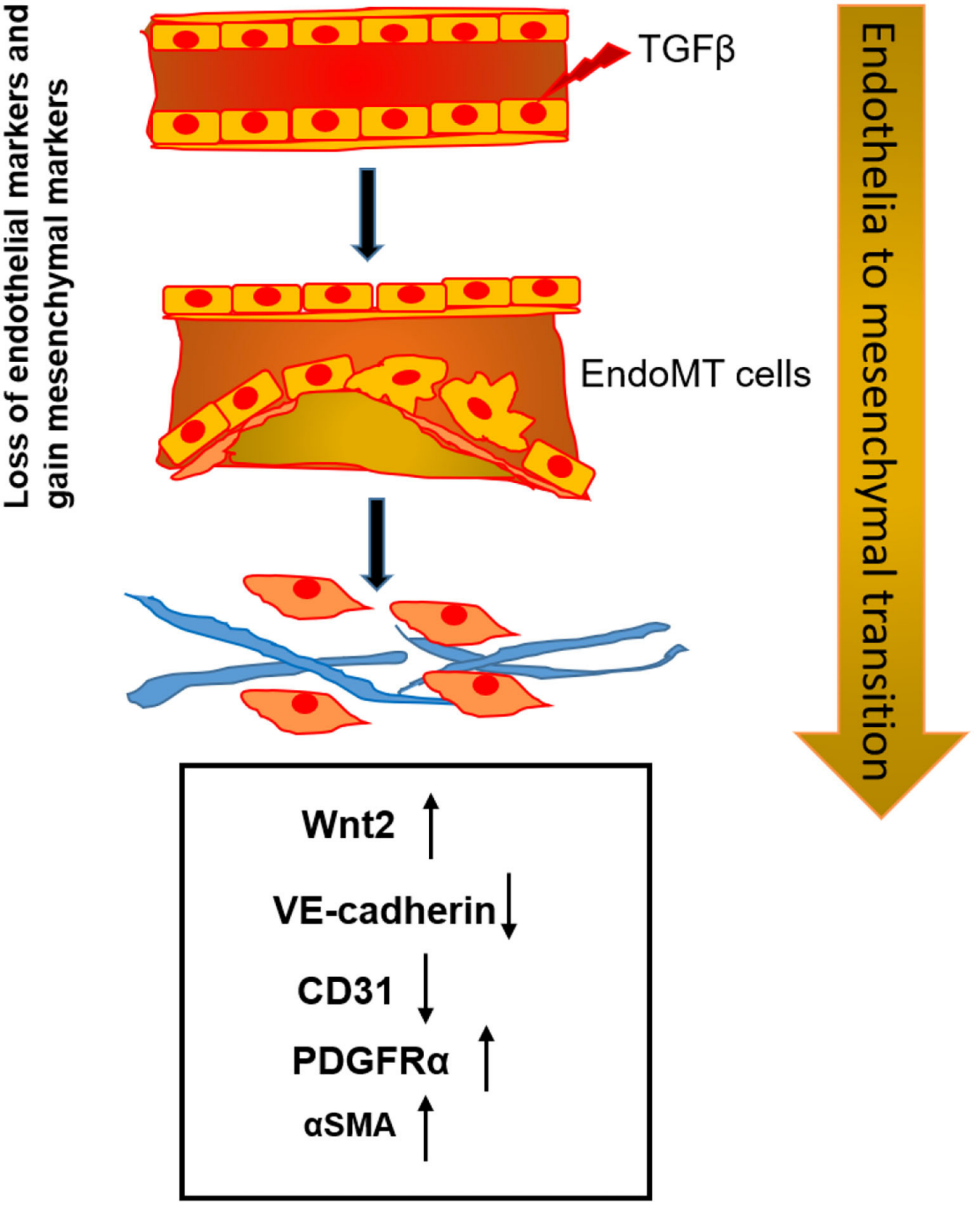
TGF-β induces EndMT in atherosclerosis. The TGF-β signaling activation initiates EndMT and induces changes in the level of the endothelial markers, VE-cadherin, CD31, and the mesenchymal makers, PDGFRα and αSMA. Suppression of endothelial TGF-β receptor expression arrest EndMT development.

EndMT is a process in which endothelial cells lose the cellular adhesion and the apical-basal polarity and gain planar polarity of spindle-shaped mesenchymal cells, which plays a pivotal role in atherosclerosis. The contribution of EndMT of arterial endothelial cells to the development of plaque in atherosclerosis has been established by *in vivo* lineage tracing experiments (5, 24). After EndMT, the accumulation of mesenchymal cells, such as myofibroblasts, smooth muscle cells, and osteoblasts, is critical to the buildup of plaques (25). EndMT-derived cells comprise almost 50% of the fibroblast population in advanced atherosclerotic lesions (26). Therefore, there is an urgent need to identify novel factors that can be targeted to attenuate EndMT and further prevent atherosclerosis. In this study, we determined that TGF-β-induced EndMT occurs in atherosclerosis and also identified Wnt2 ligand as a necessary signaling factor to enable TGF-β to induce EndMT. We also detected a high level of Wnt2 expression in atherosclerotic lesions derived from the aortic valves of LDLR^−/−^ mice fed on a HF diet. As is well-known, characteristics of EndMT include decreased expression of endothelial markers include CD31, VE-cadherin, and endothelial nitric oxide synthase (NOS3), and increased expression of mesenchymal markers including α-SMA, calponin, SM22α, and PDGFRα (27). Additional features include increased expression of EndMT-associated transcription factors such as TWIST, SMAD, ZEB, and Snai1 (28). In this study, we examined EndMT using endothelial markers, CD31, and VE-Cadherin, and mesenchymal markers, α-SMA, and PDGFRα, in HAECs before and after TGF-β treatment, and we found that Wnt2 plays an essential role in TGF-β-mediated EndMT. Further, IHC staining of atherosclerotic tissues from mice demonstrated that Wnt2 expression is much higher in atherosclerotic plaques than the surrounding normal tissues. Our findings suggest that Wnt2 may participate in atherosclerotic plaque formation through promoting EndMT as shown in Figure 6.

Previous studies indicated that EndMT is modulated by FGFR1 signaling, an important process during atherosclerosis (7). The mesenchymal phenotype of the EndMT-derived cells contributes to the atherosclerotic progression including regulating the endothelium response to oxidative stress and maintaining collagen-MMP balance (5). Further, endothelial-specific deletion of Gata4 or Twist1 reduces the lesion size in atherosclerotic mice indicating that the Gata4–Twist1–Snai1 pathway contributes to atherosclerosis (11). Furthermore, the results from *in vivo* lineage tracing experiments that employed Cdh5-CreERT2 and SCL-CreERT2 endothelial-specific Cre-lox systems crossed with an ApoE^−/−^ or LDLR^−/−^ mouse model of atherosclerosis support the direct correlation between EndMT and atherosclerosis (5, 7). Additional evidence from several different research groups further suggests that EndMT-derived cells are present in intimal plaques during the development of atherosclerosis and that the EndMT process can be driven by TGF-β signaling, oxidative stress, and hypoxia in HUAECs (29). Although these reports have demonstrated that EndMT contributes to the process of atherosclerosis, a detailed characterization of all the molecules and their underpinning mechanisms involved in this signaling pathway is still lacking. Here, we employed an *in vitro* model of TGF-β-overexpressing HAECs to further explore the novel elements and the underlying mechanism in the formation of atherosclerosis. As reported, in adults, Wnt2 enhances VSMC migration and intimal thickening *in vivo* (30); however, its role in atherosclerosis is not well-characterized. To determine whether the Wnt signaling pathway plays a critical role in TGF-β-mediated EndMT, we performed RT-PCR and immunoblotting analyses and a shRNA-mediated screen to detect the expression of Wnt family members including Wnt2, Wnt4, Wnt10B, and Wnt 11during TGF-β-mediated EndMT. We found that Wnt2 plays an essential role in TGF-β-induced EndMT. We also found that Wnt2 is highly colocalized with α-SMA in atherosclerotic lesions in LDLR^−/−^ mice fed on a high-fat (HF) diet when compared with those fed on a normal diet (ND). In Intriguingly, it will be of importance and significant impact to explore and characterize the interaction between Wnt2 ligand and atherosclerotic plaque formation. Thereby, targeting Wnt2 to prevent or attenuate plaque progression and induce regression of the EndMT process will become a novel strategy to cure atherosclerosis.

Our findings presented in this paper enhance the understanding of the context- and milieu-specific nature of EndMT using our *in vitro* model to illustrate the cell transitioning and final mesenchymal phenotype with two EndMT-inducing conditions, TGF-β treatment and TGF-β overexpression. We observed that in both EndMT induction systems, Wnt2 expression increased, indicating that the resulting mesenchymal phenotype required the expression of Wnt2 and shRNA-mediated Wnt2 silencing will attenuate the EndMT transition that is induced by TGF- β. Consistent with previous studies, atherosclerotic lesion formation was detectable in LDLR^−/−^ mice fed with a HF diet (31, 32). Furthermore, results from our immunohistochemical staining demonstrated that Wnt2 expression is significantly elevated in atherosclerotic plaques from LDLR^−/−^ mice fed with a HF diet when compared to surrounding non-atherosclerotic tissues and control tissues from mice fed with normal-chow diet. Our findings provide a new potential treatment for atherosclerosis. An interesting question is whether a Wnt2 antibody would be beneficial in treating atherosclerosis since Wnt2 is a secreted protein and can be targeted with antibodies. Wnt2 antibodies have had benefits in various cancers. A previous study reported that cancer associated fibroblasts secrete Wnt2 to promote progression in colorectal cancer (33). Also, an anti-Wnt2 monoclonal antibody induces apoptosis in malignant melanoma cells and inhibits tumor growth (34). Whether Wnt2 antibodies have a beneficial role in treating atherosclerosis will be addressed in our future studies. Nevertheless, understanding new cell signaling pathways that are involved in the initiation of atherosclerosis is pivotal to discover novel therapeutic strategies.

## Conclusions

These studies demonstrate that Wnt2 is required for TGF- β -induced EndMT and that Wnt2 expression increases in the atherosclerotic plaques in LDLR^−/−^ mice fed with a HF diet. These data present Wnt2 as a potential therapeutic target to treat atherosclerosis.

## Data Availability Statement

The raw data supporting the conclusions of this article will be made available by the authors, without undue reservation.

## Ethics Statement

The animal study was reviewed and approved by the Institutional Animal Care and Use Committee at East Tennessee State University (ETSU).

## Author Contributions

JZ, SR, SS, and MG performed experiments. DT and SS provided atherosclerotic mouse aortic samples. JZ and YJ wrote and revised the manuscript with comments and suggestions of all other authors. All authors contributed to the article and approved the submitted version.

## Funding

This work was supported by ETSU startup funding.

## Conflict of Interest

The authors declare that the research was conducted in the absence of any commercial or financial relationships that could be construed as a potential conflict of interest.

## Publisher's Note

All claims expressed in this article are solely those of the authors and do not necessarily represent those of their affiliated organizations, or those of the publisher, the editors and the reviewers. Any product that may be evaluated in this article, or claim that may be made by its manufacturer, is not guaranteed or endorsed by the publisher.

## Abbreviations

ECM: Extracellular matrix
EndMT: Endothelial-to-mesenchymal transition
FGFR1: Fibroblast growth factor receptor 1
H&E: Hematoxylin and Eosin
HAECs: Human aortic endothelial cells
HF: High fat
ND: Normal diet
LDL: Low-density lipoprotein
LDLR-/-: Low-density lipoprotein receptor
NOS3: Nitric oxide synthase
OE: Overexpression
PDGFRα: Platelet-derived growth factor receptor α
Sca1: Stem cell antigen 1
shRNA: Short hairpin RNA
α-SMA: α-smooth muscle actin (α-SMA)
SMC: Smooth muscle cells
Snai1: Zinc finger protein
TGF-β: Tumor growth factor
VE: cadherin Vascular endothelial-cadherin
WB: Western blot
WCL: Whole cell protein.
