# Supplementary material for: Wnt2 Contributes to the Development of Atherosclerosis
Source: Front Cardiovasc Med. 2021 Nov 24;8:751720. doi: 10.3389/fcvm.2021.751720 (PMC8652052; doi:10.3389/fcvm.2021.751720)
Supplement: Supplementary file 1 [file Image_1.pdf]

Supplementary figures:

Figure S1

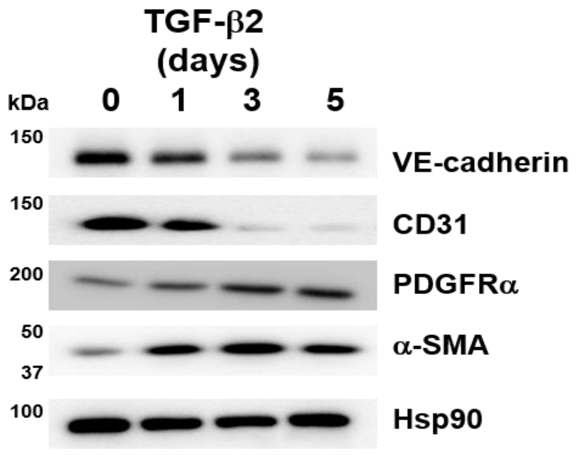

Supplementary Figure S1. TGF-β2 induces EndMT in HAECs**.** Immunoblotting for the expression of the EndMT markers VE-cadherin, CD31, PDGFRα, and α-SMA in HAECs treated with TGF-β2 for 0, 1, 3, and 5 days. Hsp90 was used as a loading control. Experiments were repeated three times.

Figure S2

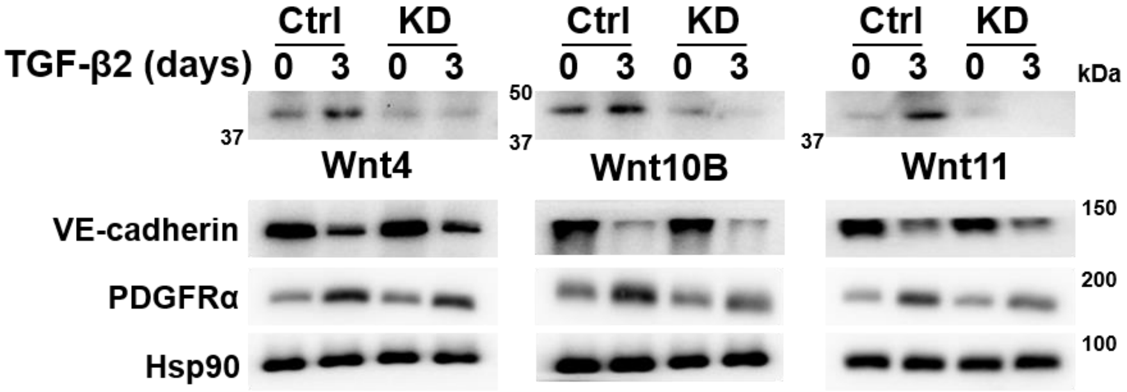

Supplementary Figure S2. The Wnt ligands are not essential for TGF-β2-mediated EndMT in HAEC cells. Immunoblotting detection of the expression of the EndMT markers VE-cadherin and PDGFRα in control and the indicated Wnt ligand KD HAEC cells with a 3-day TGFβ treatment. Hsp90 was used as a loading control. Experiments were repeated three times.
